# Carbon-Supported Hyperbranched Polyethyleneimines: Exploring into Polyamine/Anion Interactions to Design Efficient Polymer-Based Energy and Scavenger Materials

**DOI:** 10.3390/polym17060786

**Published:** 2025-03-15

**Authors:** Antonio Peñas-Sanjuán, Celeste García-Gallarín, María L. Godino-Salido, Rafael López-Garzón, Michele Melchionna, Manuel Melguizo

**Affiliations:** 1 Department of Inorganic and Organic Chemistry, Faculty of Science, University of Jaén, 23071 Jaén, Spain; cgarcia@ujaen.es (C.G.-G.); mlgodino@ujaen.es (M.L.G.-S.); rlopez@ujaen.es (R.L.-G.); mmelgui@ujaen.es (M.M.); 2 Department of Chemical and Pharmaceutical Sciences, Center for Energy, Environment and Transport Giacomo Ciamician and INSTM Trieste Research Unit, University of Trieste, 34127 Trieste, Italy; melchionnam@units.it

**Keywords:** hyperbranched polyethyleneimine, activated carbon, energy materials, anion scavengers, anion-complexing mechanism

## Abstract

The anion-complexation mechanism and anion-adsorption capacity of a hybrid material based on hyperbranched polyethyleneimine (HBPEI) covalently bonded onto an activated carbon (AC) is presented. The anion-scavenger behavior of this hybrid material toward CrO_4_^2−^, PO_4_^3−^, AsO_4_^3−^ and HgCl_4_^2−^ was explored by direct potentiometric and adsorption measurements, which revealed a novel approach to predict the interactions between the supported polymeric complexing units and the different anions. The results were analyzed by considering the reactivity data of the HBPEI/anion (HBPEI free in solution) and AC-HBPEI/anion systems. The results corroborated that the AC-HBPEI hybrid material is an excellent anion-complexing material, whose anion adsorption ability is defined by the complexing properties of the HBPEI molecules toward the anions. This assessment provides a straightforward tool to determine the type and strength of the interactions involved in supported polymer-based/anion systems, which can provide valuable information for predicting and designing efficient energy and scavenger materials.

## 1. Introduction

The anion-binding molecular structures are of growing interest due to their wide implications in energy [1,2,3], biomedical [4,5] and environmental applications [6,7]. Consequently, extensive research has focused on broadening the understanding of supramolecular anion binding, through both the development of innovative complexing agents and the investigation of interaction mechanisms [8,9,10,11,12]. Traditionally, most research studies were focused on the development of anion receptors based on single molecules with a defined affinity toward a specific metal anion [13,14,15,16]; however, during the last decade, steady research based on the design of polymer-based anion receptors, with strong and selective anion-binding properties, has emerged as a promising strategy [17,18].

Among the vast diversity of existing polymers, recent studies have shown that polyamines are one of the most successful receptors for ion-binding in aqueous media, via electrostatic and hydrogen bond interactions, due to the versatile nature of the amino groups [19,20,21,22,23,24,25,26,27,28]. Bearing in mind such behavior, the use of water-soluble polyamines covalently bonded to solid supports can provide efficient anion scavengers with outstanding properties. In general, polyamines, and particularly polyethyleneimines (linear and branched) [29], have been studied as complexing agents for heavy metals in aqueous solutions [30]; however, a detailed investigation of their anion-complexing capacity is essential, given the limited research on this topic [31,32,33].

**Figure 1:**
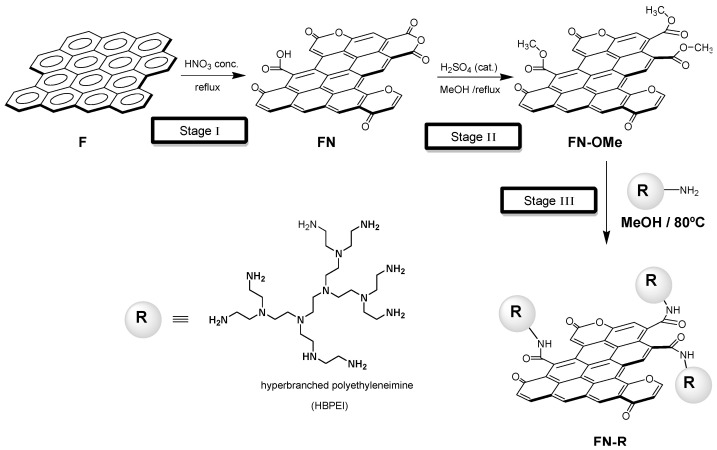
Synthetic strategy to obtain a hybrid activated carbon with grafted polyethyleneimine (HBPEI).

We recently reported results on the preparation of a hybrid carbon material (labeled FN-HBPEI), based on covalently bonded hyperbranched polyethyleneimine (HBPEI) functions, obtained through a procedure consisted of three reaction steps (see Figure 1): (i) oxidation of a commercial activated carbon (labeled F) with HNO_3_, rendering the intermediate oxidized carbon, FN; (ii) esterification of carboxylic groups introduced in FN to transform them into methyl esters, rendering the FN–OMe material; and (iii) bonding the HBPEI molecules, through amide linkages, via a reaction between the carboxylic esters of FN-OMe and the primary amino groups of HBPEI, obtaining the hybrid material FN-HBPEI [34].

**Figure 2:**
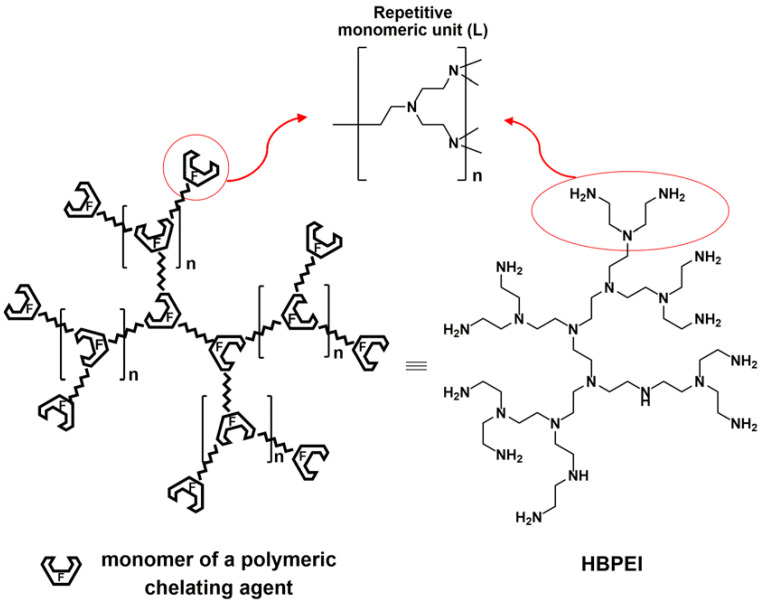
Polychelatogen structure of hyperbranched polyethyleneimines (HBPEIs).

The interest in this hybrid material lies in the polyaminic structure of the bonded HBPEI, which acts as a polychelatogen agent formed by the multiple complexing centers (Figure 2), acting as selective “amplifier” functions on the AC surface [35]. This previous study showed that the adsorption capacity of FN-HBPEI (see Figure 1) toward heavy metals is clearly related to the complexing abilities of HBPEI molecules in aqueous solution. As a consequence, the HBPEI molecules grafted on the carbon surface were responsible for the enhanced absorption capacity and selectivity towards several metal ions [35].

Herein, we report a detailed study concerning the ability of the aforementioned polymer-based hybrid material (FN-HBPEI) to function as an anion scavenger. This study analyzes the adsorption capacity and the nature of the interactions established between the polymeric complexing units and different anions with electrochemical (CrO_4_^2−^, PO_4_^3−^) [36,37,38,39] and environmental interest (AsO_4_^3−^ and HgCl_4_^2−^) in aqueous solution [40,41,42,43]. The obtained results expand the knowledge about the processes and interactions involved in anion/FN-HBPEI systems, pointing out some limitations that hinder conformational changes in the grafted-HBPEI molecules within the FN-HBPEI porous structure. As such, this study provides remarkable insights regarding the use of potentiometric measures to understand the nature and strength of the interactions involved in hybrid systems based on polymeric structures and anions, with potential interest for energy applications [36,37,38,39].

## 2. Materials and Methods

**Materials**. The hybrid carbon material (FN-HBPEI) was synthesized following a previously reported method [34]. HBPEI (Mn 600), NaH_2_PO_4_, KH_2_AsO_4_, K_2_CrO_4_, HgCl_2_, KCl, KOH and HCl were purchased from Merck-Aldrich (Madrid, Spain) and used without further purification.

**Preparation and Characterization of FN-HBPEI**. The hybrid FN-HBPEI was prepared from HBPEI (Mn 600) and a commercial activated carbon, F (Filtracarb—SKI 8 × 30 from CPL Carbon Link (Wigan, UK); elemental analysis N (0.36%), C (90.34%), H (0.22%), O (8.83%) and pHpzc = 5.50). The procedure consisted of three reaction steps: (i) oxidation of F with HNO_3_ (rendering FN); (ii) esterification of carboxylic groups introduced after oxidation of F to transform them into methyl esters (rendering FN-OMe); and (iii) bonding of HBPEI (Mn 600) to the AC, through amide linkages obtained by the reaction of the carboxylic esters on the carbon surface with the primary amino groups of the HBPEI (rendering FN-HBPEI) [34]. The hybrid FN-HBPEI had a high N content according to combustion elemental analysis [N (9.22%), C (73.95%), H (3.22%), O (13.36%)], which correspond to 0.37 mmol per gram of grafted HBPEI (Mn = 600, DP = 14). The preservation of the covalent structure of polyethyleneimine, in the hybrid FN-HBPEI, was confirmed by *ss*-^13^C-NMR (relevant signals around 50 ppm due to N-CH_2_-CH_2_-N groups and 165–170 ppm range due to carboxamide groups). In addition, the linkage through amide bonds was confirmed by XPS analyses (peak at 399.6 eV in the N1s region due to nitrogens of alkylamino functions and a shoulder at 401.7 eV in the N1s region due to amide nitrogens) and compared with those of model compounds. The point of zero surface charge of the hybrid FN-HBPEI was at pH 9.07, in coherence with the amine functionalization at the surface. FN-HBPEI showed a very low specific surface area (by the BET equation), of only 77 m^2^g^−1^, which when compared with that of its direct precursor (esterified carbon -FN-OMe) of 661 m^2^g^−1^, indicates blockage of the porous system (of the carbon support) due to the fixation of the polyamines at the internal surface of pores (see Supporting Information). In addition, TGA of FN-HBPEI revealed a weight loss from room temperature to 200 °C caused by the moisture/bound water present in the materials. In addition, between 200 and 400 °C, a weight loss of 13.3% was observed for FN-HBPEI, attributed to HBPEI degradation.

Textural characterization was carried out by N_2_ (at 77 K) and CO_2_ (at 273 K) adsorption. The adsorption isotherms were obtained using Micromeritics ASAP 2020 equipment (Granada, Spain), and textural parameters were derived by applying the BET and Dubinin–Radushkevich equations to the experimental isotherms.

The XPS spectra of the HBPEI and FN-HBPEI were registered with an ESCA 5701 instrument (Physical Electronics) (Málaga, Spain), using the Mgk_α_ 300w 15 kV radiation of twin anodes in the constant analyzer energy mode, with pass energies of 187.85 eV (for the survey spectrum) and 29.35 eV (for narrow atomic ranges). The pressure of the analysis chamber was maintained at 4 × 10^−9^ Torr. The binding energy and the Auger kinetic energy scale were regulated by setting the C1s transition at 284.6 eV. The accuracy of BE values was ±0.2 eV.

The NMR spectra were measured in a Bruker Advance 500 instrument (Jaén, Spain) equipped with a standard-bore 11.74 T superconducting magnet operating at 500.13 MHz for ^1^H and at 125.76 MHz for ^13^C, equipped with two channels, magic angle spinning (MAS) and a broadband probe (Bruker X/H CP-MAS) for 4 mm diameter sample rotors. All the NMR experiments were performed in the direct polarization (DP) mode. A total of 33,000 transients were acquired from a mixture of carbon material–silica gel 70:30 (*w*/*w*) with MAS at 12 kHz; a pulse width (nominal) of 17° was used with a recycle delay of 2 s, and no proton decoupling was applied.

Thermogravimetric analysis (TGA) was carried out in a SHIMADZU model TGA-50H (Málaga, Spain) under a nitrogen atmosphere with a flow of 50 mL/min, in a temperature range of 25 °C to 950 °C with a step of 10 °C/min

**Molecular modeling of HBPEI and HBPEI-nH^+^ systems**. Optima geometries of HBPEI molecules (non-protonated and with several protonation degrees) were calculated from the conformations corresponding to the global minimum energy by using the MM2 method and applying the Polak–Ribiere algorithm (ChemBats3D, CambridgeSoft Corp., Cambridge, MA, USA) [44]. The geometries were optimized without any constraints, allowing all atoms, bonds and dihedral angles to change simultaneously. The final RMS gradient was less than 0.001 kcal mol^−1^ Å^−1^ for all the minimized structures. Extended Hückel molecular orbital (EHMO) analyses for idealized geometries were performed with the HYPERCHEM software Release 8.0 [45].

**Potentiometric studies**. Acid–base titrations were carried out in an automatic potentiometric titrator (Methrom 765 Dosimat, Madrid, Spain) equipped with a Metrohm Glass electrode and a Metrohm 713 pH meter for pH measurements [46]. The system was calibrated as a hydrogen concentration probe by titrating known amounts of HCl with CO_2_-free NaOH solutions and determining the equivalent point by Gran’s method [47]. This allows for determining the standard potential, E_o_, and the ionic product of water (pK_w_ = 13.83, 0.10 M KCl) at 298.0 K. At least three potentiometric titrations (about 100 data points each) were performed for all the systems (see below) in the 2.5–10.5 pH range [48]. The Hyperquad software (version 2013) [49,50] was used to calculate the equilibrium constants from the corresponding emf data.

(a) ***HBPEI/H^+^ and HBPEI/anion systems***

The protonation equilibria of the HBPEI molecules, defined by the formation of different *HL* species (H = acid protons and L = complexing triethylenetriamine units of HBPEI) and their corresponding constants, were obtained from the pH data of the potentiometric titration of an HBPEI water solution with ionic strength 0.1 M KCl, at 298.0 K, as already reported [35], considering HBPEI molecules as constituted by the repetition of triethylenetriamine structural units (L= –NH(CH_2_)_2_–N[–(CH_2_)_2_NH_2_]–(CH_2_)_2_–) (see Section 3). In all titrations, the concentration of HBPEI (as a concentration of L units) was 10^−3^ M.

The reactivity of the HBPEI/anion systems was studied by analyzing the pH data obtained from potentiometric titrations of 0.1 M KCl water solutions of different HBPEI/anion mixtures. The molar ratios were 1/1, the temperature 298.0 K and the titrant a 0.1 M KOH solution. In all the experiments, the concentration of the triamine monomeric unit (L) and anion was 10^−3^ M.

(b) ***FN-HBPEI/H+ and FN-HBPEI/anion systems***

Protonation equilibria of FN-HBPEI and their corresponding constants were determined from the pH data obtained in the potentiometric titration of an FN-HBPEI water suspension (0.1 g of FN-HBPEI, which had 0.18 mmol of grafted triethylenetriamine units, L) with ionic strength 0.1 M KCl, at 298.0 K.

For the analysis of the reactivity of FN-HBPEI/anion systems, a suspension of 0.1 g of FN-HBPEI (containing 0.18 mmol of grafted triethylenetriamine units, L) in 40 mL of 0.1 M aqueous KCl solution was prepared. Then, pH was adjusted to ca. 2.5 by adding HCl aqueous solution [35]. After 48 h of equilibration time under a N_2_ atmosphere, the anion was added to the solution, and we kept stirring for 48 h until equilibrium was reached. Subsequently, the suspension was titrated with 0.1 M NaOH up to pH 10.5. An equilibration time of 1800 s elapsed between each titrant addition (0.03 mL). In all the experiments, an HBPEI (as L units)/anion molar ratio of 1/1 was used. The Hyperquad software (version 2013) [50] was used to calculate the equilibrium constants from the emf data.

**Adsorption Measurements**. Water solutions of NaH_2_PO_4_, KH_2_AsO_4_, K_2_CrO_4_ and HgCl_2_ salts were used in the adsorption experiments of anions on FN-HBPEI. The equilibration times were previously determined by means of independent experiments. For this purpose, flasks containing 25 mL of 10^−3^ M anion solution and 25 mg of adsorbent were prepared and maintained under stirring. Then, the anion concentration was measured at different times. Once the equilibrium times were determined, the adsorption isotherms of anions were obtained at 298.0 K. Typically, 25 mg of adsorbent (FN-HBPEI) was added to a 100 mL plastic flask containing 25 mL of anion solution. The anion concentration was varied between 6 × 10^−5^ and 1 × 10^−3^ M. The required initial pH of the anion solutions (6.7 for PO_4_^3−^, 6.7 for AsO_4_^3−^, 7.5 for CrO_4_^2−^ and 3.0 for HgCl_4_^2−^) was obtained by adding HCl or NaOH solutions. The anion concentration at the equilibrium was determined by means of ICP-mass (in the case of PO_4_^3−^, AsO_4_^3−^ and CrO_4_^2−^) and UV (for HgCl_4_^2−^) measurements. In addition, adsorption experiments toward each particular anion on the original activated carbon, F (pristine AC), were performed in order to compare the maximum anion retention capacity (X_m_) on F against the X_m_ values obtained on FN-HBPEI; such experiments rendered X_m_ values lower than 0.01 mmol anion g^−1^ for carbon F. Blank experiments were also performed to verify that neither the ligand nor the metals were adsorbed by the plastic flasks.

**Conductimetric measurements**. Conductometric titration was carried out with an automatic potentiometric titrator (Metrohm 702 SM Titrino, Madrid, Spain) endowed with a conductivity meter (Metrohm 712 SM Titrino, Madrid, Spain). The data were acquired by the titration of 50 mL of HgCl_4_^2−^ water (double-distilled) solution. The initial concentration of HgCl_4_^2−^ was 10^−3^ M, and the pH was adjusted to 3. Then, the suspension was titrated with a 10^−3^ M solution of HBPEI up to an HBPEI/HgCl_4_^2−^ molar ratio of 6/1. An equilibration time of 900s elapsed between each titrant addition (0.03 mL) to allow the stability of the pH and conductance values [46]. Additionally, the conductimetric titration of a blank consisting of 50 mL of distilled water with the same titrating solution was also carried out under the same conditions. The differences between the values of both titrations were used to obtain information about complex formation in an HBPEI/HgCl_4_^2−^ system.

## 3. Results and Discussion

The assumption that the complexing properties and binding mechanisms, of HBPEI/metal ions and FN-HBPEI/metal ion systems, are defined by the triethylenetriamine complexing units (L) of HBPEI molecules was previously proved in our laboratory [35]. However, this model has not been previously studied for anion species, since protonated polyamines are complexing agents capable of binding anions by forming (poly)cation-(poly) supramolecular species [48]. In this study, the anions were selected on the basis of two current needs: (i) Anions’ application in energy materials (CrO_4_^2−^, PO_4_^3−^), particularly in electrolytes [32,36,37,38,39]. The importance of anions in electrolytes is often underestimated, despite their significant impact on battery performance and stability [3], where a thorough understanding of anion chemistry, including the bonding and mechanism interaction, is crucial. (ii) Removal of anions with marked polluting activity (AsO_4_^3−^ and HgCl_4_^2−^) due to their high environmental impact and complex treatments [33,40,41] from the environment. Thus, firstly the reactivity of the HBPEI toward each particular anion, in aqueous solution, was analyzed by potentiometric titrations and conductimetry to determine the strength and the mechanism of the interactions. Then, the adsorption behavior of the FN-HBPEI was studied, and the results were analyzed by taking into consideration the HBPEI reactivity data. Finally, in light of these results, a reactivity study of FN-HBPEI/anion systems was undertaken to ascertain the effect of grafting HBPEI onto activated carbon on its anion complexation capabilities [35].

### 3.1. Complex Formation in HBPEI/Anion Systems

The data obtained from potentiometric titrations of HBPEI/anion systems were analyzed considering that HBPEI is constituted by repeating units (functional unit = L) of triethylenetriamine (L= –NH(CH_2_)_2_–N[–(CH_2_)_2_NH_2_]–(CH_2_)_2_–). These units are capable of acting as independent units with a maximum of three protons [29]. By assuming this hypothesis, the results were successfully fit according to the equilibria A*^a^*^−^ + L + *_m_*H^+^ ⇆ (ALH*_m_*)^(*a*−*m*)−^ [20]. However, these data do not provide any information about the basic positions of the interacting partners in which the *m* protons are distributed. In the case of HgCl_4_^2−^, which is not protonated in the studied pH range (2.5–10.5), the complexation equilibria can undoubtedly be described as the binding of the anion A^a−^ to the m-protonated ligand. On the contrary, in the case of CrO_4_^2−^, PO_4_^3−^ and AsO_4_^3−^, which undergo protonation in a wide pH range [51], additional experimental data are needed to establish the proton distributions in the (ALH*_m_*)^(*a*−*m*)−^ complexes. In the case of single polyamines, ^1^H and ^13^C NMR measurements have shown [23,27] that the relative basicities of the free ligands and anions are maintained in anion/ligand mixtures. In this work, analysis of the ^1^H and ^13^C NMR signals of the HBPEI methylene groups was prevented as their interpretation was not feasible. Therefore, it was assumed that the protonation of both anions and functional units takes place according to their relative basicities. In accordance with this assumption, the analysis of the potentiometric data from the HBPEI/anion systems provided the equilibrium and stability constants shown in Table 1.

**Figure 3:**
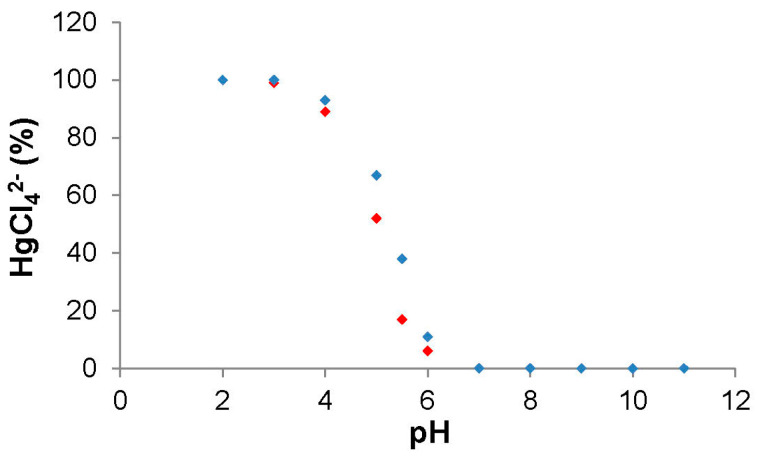
Percentages of HgCl_4_^2−^ at different pH values, calculated from: (A) the species distribution plot obtained by potentiometric titration in the HBPEI/HgCl_4_^2−^ system (red points; see Supporting Information Figure S6); and (B) UV spectra of an L/HgCl_4_^2−^ mixture in aqueous medium ([L]/[HgCl_4_^2−^] = 1/1, [L] = 5 × 10^−5^ M) in the 2–11 pH range (blue points).

In general, the stability constants show the formation of notably stable cation (*LH_n_^n+^*)–anion (*AH_m_*^(*a*−*m*)−^) complexes. In particular, note the very high stability constants of the complexes formed with HgCl_4_^2−^ in the whole range of pH values, pointing to a double complexing mechanism depending on the pH, i.e., the formation of both HgCl_4_^2−^ complexes with protonated L units (non-covalent interaction) and Hg(II) complexes with non-protonated L units (covalent interaction). This double-complexing behavior was assessed through the analysis of additional data. Firstly, the titration of an L/HgCl_4_^2−^ mixture in aqueous solution ([L]/[HgCl_4_^2−^] = 1/1, [L] = 5 × 10^−5^ M) in the 1–13 pH range was performed. This was followed by UV measurements, recording the spectra in the 200–300 nm range, from which the concentrations of HgCl_4_^2−^ were obtained (Figure 3). The data analysis shows that the UV band intensity does not change in the 1–3 pH range, which is justified because in such a pH range, HgCl_4_^2−^ is complexed through non-covalent interactions, as HgCl_4_^2−^/LH_n_^n+^ species. Subsequently, as the pH increases above 3, the intensity of the HgCl_4_^2−^ band decreases gradually until its total extinction at pH = 7. This is due to the conversion of HgCl_4_^2−^ to HgCl_2_ and, consequently, the formation of Hg^2+^/LH_n_^n+^ species. This result is in good agreement with the data obtained from the potentiometric study. Thus, the species distribution plot for the HgCl_4_^2−^/HBPEI system (see Supporting Information Figure S6) reveals the formation of HgCl_4_^2−^/HBPEI species up to pH = 3; then, the Hg^2+^/HBPEI species are gradually formed as the pH increases up to their total conversion at pH 7.

**Figure 4:**
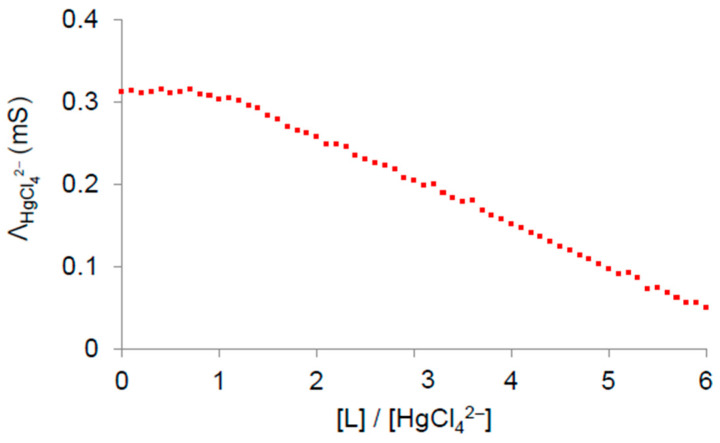
Conductometric measurements at different [L]/[HgCl_4_^2−^] molar ratios.

Additional evidence of the observed HgCl_4_^2−^/HBPEI complexes, based on protonated L units, was obtained from molar conductivity measurements, Λ (referring to HgCl_4_^2−^, see Section 2), of L/HgCl_4_^2−^ mixtures at pH = 3 and molar ratios between 0 and 6 (Figure 4). The data analysis showed a change in the slope of the Λ vs. [L]/[HgCl_4_^2−^] plot at a value of [L]/[HgCl_4_^2−^] = 1, which points to the immobilization of HgCl_4_^2−^ anions due to the formation of LH_n_^n+^/HgCl_4_^2−^ complexes with 1:1 stoichiometry. Moreover, the sharp change in the slope suggests the formation of very stable complexes, which is in good agreement with the potentiometric results (Table 1). Thus, these data support the complexing model suggested from the potentiometric data of the HgCl_4_^2−^/LH_n_^n+^ system at low pH values.

Regarding, HBPEI/CrO_4_^2−^, HBPEI/PO_4_^3−^ and HBPEI/AsO_4_^3−^ systems, our analysis was carried out considering electrostatic and hydrogen bond interactions as the main contribution to the stability of “host (*H_n_L^n+^*)*-* guest (*AH_m_*^(*a-m*)−^)” complexes. In principle, electrostatic interaction of opposed charges is expected to be the main contributor to complex formation, even though this requires releasing of water molecules from the hydration spheres of the anions. This release is enthalpically unfavorable in a solvent with a high dielectric constant, such as water, but it is largely favored due to the significant increase in entropy in the system [12]. In addition to the electrostatic interactions, the formation of hydrogen bonds, between the polyaminic receptor (HBPEI) and anions, also contributes to strengthening the anion/HBPEI associations.

The analysis of the HBPEI/anion systems show the presence of L/anion species with 1:1 and 2:1 stoichiometries for the cases of CrO_4_^2−^, PO_4_^3−^ and AsO_4_^3−^. It is particularly worth noting the species formed between the monoprotonated ligand (HL_2_^+^) and the deprotonated anions, since the formation of similar complexes with 2:1 stoichiometries, from the above anions, was not observed for analogous non-polymeric polyamine ligands [27]. This suggests that the quasi-dendritic structure of the HBPEI molecule provides a cooperative interaction of two (monoprotonated) functional units with the anions. In the case of HL_2_^+^/anion species (anion = PO_4_^3−^, AsO_4_^3−^ and CrO_4_^2−^), the hydrogen bond contributions come from N-H····^−^O and N-H^+^····^−^O interactions. Thus, the significantly higher stability of [HL_2_(AsO_4_)]^2−^ and [HL_2_(PO_4_)]^2−^ species than [HL_2_(CrO_4_)]^−^ is due not only to the higher charge of the anions (+3), but also to the larger hydrogen bond contribution, since the protonation enthalpies of AsO_4_^3−^ and PO_4_^3−^ are larger than that of CrO_4_^2−^ [44]. Regarding the species with 1:1 stoichiometry, it is worthy to note that the arsenate and phosphate anions have similar complexing behaviors, with both leading to HL^+^ and H_2_L^2+^/HA^2−^ species. Therefore, the stability constants for both anions increase with the charge of the cation. Moreover, the high values of the stability constants reflect that not only do electrostatic attractions contribute to the complex formation but also hydrogen bonds through N-H····^−^O, N-H^+^····^−^O, N-H^+^····OH, N-H····OH and HN····HO interactions. In the case of chromate, note that HCrO_4_^−^ interacts with H_2_L^2+^ more strongly than CrO_4_^2−^, contrary to the expected behavior based on electrostatic considerations. This fact is likely due to the greater ability of HCrO_4_^−^ to act as hydrogen donor, since HN····HO hydrogen bonds are effective at modifying the order of stability expected on the basis of single electrostatic considerations [27].

### 3.2. Adsorption Studies of Anions

Adsorption studies of FN-HBPEI/PO_4_^3−^, FN-HBPEI/AsO_4_^3−^, FN-HBPEI/CrO_4_^2−^ and FN-HBPEI/HgCl_4_^2−^ systems, in aqueous solutions, were carried out to gain insights into the effect of the grafted-HBPEI molecules on the surface properties of the hybrid material [52,53]. For this purpose, the adsorption capacity of FN-HBPEI, toward the mentioned anions, was compared with the previously assessed reactivity data obtained from the HBPEI/anion systems in aqueous solutions.

The adsorption isotherms were obtained at pH values at which the HBPEI reactivity toward the anions was maximum. These pH values were those corresponding to the minimum amount of non-bonded anion (see the Section 2) obtained from the species distribution plots of the HBPEI/anion systems. The chemical nature of the adsorbed anions was determined by using the XPS spectra of the FN-HBPEI/anion species, in order to check for possible changes in the anion oxidation state, induced by the adsorption mechanism. The XPS spectra showed that none of the anions changed its oxidation state upon adsorption.

**Figure 5:**
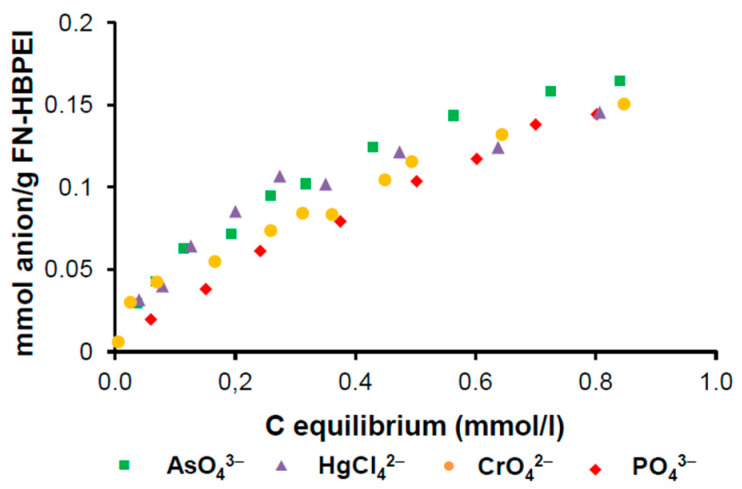
Adsorption isotherm of FN-HBPEI/anion systems.

The good fittings of the anion adsorption isotherms (Figure 5) to the Langmuir model are consistent with the existence of a predominant adsorption mechanism in all systems. This suggests the existence only of an anion-complexing function on the surface of the adsorbent, which agrees with the proposed model defining the FN-HBPEI surface covered with covalently bonded HBPEI molecules [35]. Consequently, the only available functions, with complexing properties on the FN-HBPEI surface, are the triamine units of the grafted-HBPEI molecules, whereas most internal oxygen functions of the carbon (carboxyl, lactone, phenol or methyl ester) are inaccessible. The fit of the adsorption isotherm data to the Langmuir equation rendered the maximum adsorption capacity values (X_m_) of all anions (AsO_4_^3−^ = 0.16 mmol g^−1^, PO_4_^3−^ = 0.13 mmol g^−1^, CrO_4_^2−^ = 0.15 mmol g^−1^ and HgCl_4_^2−^ = 0.16 mmol g^−1^). These values reveal that the hybrid material (FN-HBPEI) has a high adsorption capacity toward the mentioned anions in aqueous solution.

In order to assess the complexing ability of FN-HBPEI toward the anions, KOH titrations of FN-HBPEI/anion mixtures were performed in the same experimental conditions as their HBPEI/anion counterparts. The results showed a very good fit with the complexation model displayed in Table 2, which indicates that the only available functions in FN-HBPEI are the L-units of the HBPEI molecules. The data revealed that the complexes formed between PO_4_^3−^, AsO_4_^3−^, CrO_4_^2−^ and FN-HBPEI are similar (species distribution and stability constants) to their analogous complexes formed with non-grafted polyamine (HBPEI molecules in solution). This suggests that in these cases, adsorption takes place through a complexation mechanism with partially protonated L-units. Nevertheless, the FN-HBPEI/HgCl_4_^2−^ system has a different species distribution at low pH values, with respect to that observed in the HBPEI/HgCl_4_^2−^ system. This fact is probably due to structural restrictions in the fully protonated L units of the grafted-HBPEI molecules, hindering the conformational changes needed for complexation, when the L units are highly charged.

This hypothesis was considered by taking into account the textural features of FN-HBPEI (specific surface area, total micropore volumes and average pore widths determined from N_2_ and CO_2_ adsorption isotherms, see Table 3) and the molecular dimensions of the HBPEI molecules under different protonation degrees, which were calculated from the conformations corresponding to the global minimum energy by using the MM2 method and applying the Polak–Ribiere algorithm (see Table 4). Thus, bearing in mind that the surface of FN-HBPEI consists of a homogeneous distribution of grafted-HBPEI molecules [35], the proximity of these molecules will constrain their molecular expansion at high protonation degrees. This is particularly relevant in the formation of H_3_L^3+^ species, as can be observed in Table 4. Moreover, molecular expansion’s restriction is even more important for HBPEI molecules grafted onto small pores. The data in Table 3 show that the parent active carbon, F, has a mean micropore size, L_0_(N_2_), in which the neutral HBPEI (L in Table 4) can be grafted. In addition, this sample has small mesopores, which can be occupied by the HBPEI molecules [54]. A dramatic decrease in the micropore volume measured by nitrogen, V_0_(N_2_), and by carbon dioxide, V_0_(CO_2_), is observed after HBPEI is grafted. The decrease in V_0_(N_2_) means that some of the grafted molecules are occupying large micropores and small mesopores. Indeed, there is no available space in these pores for H_3_L^3+^ formation, according to the data in Table 4. Therefore, for the above reasons, the formation of [H_3_L(HgCl_4_)]^+^ (main species at pH = 3, c.a. 75%) and [H_2_L(HgCl_4_)] complexes (also formed, c.a. 25%) on FN-HBPEI is probably hindered.

Considering these data, it can be expected that when the HBPEI molecules are grafted onto activated carbon, their chelating abilities toward PO_4_^3−^, AsO_4_^3−^ and CrO_4_^2−^ are preserved. Thus, the maximum adsorption capacities of FN-HBPEI, for these anions, are related to the complexation abilities of the grafted-HBPEI molecules. This is because HL^+^ and H_2_L^2+^ species, which are the potential complexing units at the studied pH values (6.7 for PO_4_^3−^, 6.7 for AsO_4_^3−^ and 7.5 for CrO_4_^2−^), have no severe stereochemical restrictions. Nevertheless, in the case of HgCl_4_^2−^ at pH 3, the FN-HBPEI surface consists of a rigid positively charged HBPEI molecular distribution, which hinders the diffusion of HgCl_4_^2−^ into the grafted-HBPEI molecules, limiting the complexation by the H_3_L^3+^ units (the main species at pH 3).

**Figure 6:**
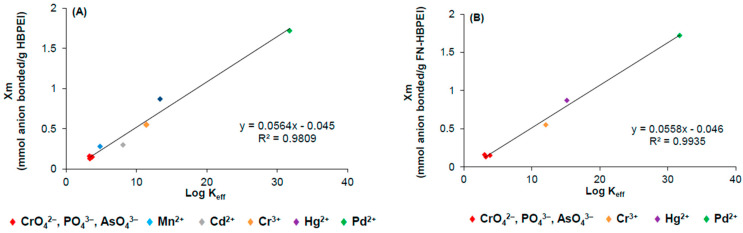
Correlation between log K_eff_ and X_m_ values for (**A**) HBPEI/ion and (**B**) FN-HBPEI/ion systems. Value corresponding to HgCl_4_^2−^ species was excluded.

This was assessed by comparing the effective stability constant, K_eff_, with the corresponding maximum adsorption capacities, X_m_, of FN-HBPEI. For this purpose, K_eff_ was obtained from the species distribution plots using the following equation:

(1) $$K_{eff}=\sum \left[H_{m}AH_{n}L\right]/\sum \left[H_{i}A\right]\times \sum \left[H_{j}L\right]$$

where [H_m_AH_n_L] is the concentration of the different complex species, [H_i_A] the protonated anion species and [H_j_L] the ligand species (charges are omitted for simplicity) [55]. Figure 6 shows a good linear relationship between X_m_ and log K_eff_, not only for the anions of this study but also for several cations previously reported [35]. The relevance of this plot is that it supports the above statement, i.e., the adsorption on the hybrid material is defined by a complexation mechanism directed by the HBPEI molecules. The similar X_m_ values for PO_4_^3−^ (0.13 mmol/g adsorbent), AsO_4_^3−^ (0.16 mmol/g adsorbent) and CrO_4_^2−^ (0.15 mmol/g adsorbent) are consistent with the very close values of their corresponding K_eff_ in aqueous solution, at the studied pH values. Nevertheless, in the case of the HgCl_4_^2−^/FN-HBPEI system, the adsorption capacity (0.16 mmol/g adsorbent) is very small compared to its much higher stability constant (Log K 8.56). This is consistent with the hypothesis that the complexation mechanism of HgCl_4_^2−^, at the required low pH values, is hindered due to the stereochemical restrictions, which limit the formation of H_3_L^3+^ species.

## 4. Conclusions

A straightforward approach to predict, through direct potentiometric measurements, the complexing properties and adsorption capacity of polymer-based hybrid materials toward particular anions has been proposed. The results obtained from the reactivity studies reveal that the adsorption capacity of the FN-HBPEI hybrid material depends on the complexing properties of the grafted-HBPEI molecules toward the selected anions. As a result, a very good agreement between the stability constants, K_eff_, for each anion and their maximum adsorption capacities on FN-HBPEI was found. In addition, the results show that the complexing capacity of the grafted-HBPEI molecules toward HgCl_4_^2−^ is limited when the complexing unit, L, is fully protonated, which is ascribed to structural restrictions resulting from electrostatic repulsive forces between the very close clusters of HBPEI molecules on the FN-HBPEI surface. Interestingly, the collection of potentiometric studies on HBPEI/anion and FN-HBPEI/anion systems demonstrate that simply by using potentiometric measurements, we can determine the type and strength of the interactions established between polymer-based hybrid materials and anions, which can offer a very useful tool to predict the efficiency of complex hybrid materials, such as polymer electrolytes in batteries, where the interaction with anions is crucial for the system’s efficiency.

## Figures and Tables

**Table 1:** Stability constants of HBPEI/anion complexes in solution (0.1 M KCl, 298.0 K).

Equilibrium	Log *K* *
HL_2_^+^ + AsO_4_^3−^ ⇆ [HL_2_(AsO_4_)]^2−^	7.02 (6)
HL^+^ + HAsO_4_^2−^ ⇆ [HL(HAsO_4_^2−^)]^−^	2.98 (8)
H_2_L^2+^ + HAsO_4_^2−^ ⇆ [H_2_L(HAsO_4_)]	3.70 (4)
HL_2_^+^ + PO_4_^3−^ ⇆ [HL_2_(PO_4_)]^2−^	7.85 (6)
HL^+^ + HPO_4_^2−^ ⇆ [HL(HPO_4_^2−^)]^−^	3.12 (6)
H_2_L^2+^ + HPO_4_^2−^ ⇆ [H_2_L(HPO_4_)]	3.84 (3)
HL_2_^+^ + CrO_4_^2−^ ⇆ [HL_2_(CrO_4_)]^−^	4.38 (6)
HL^+^ + CrO_4_^2−^ ⇆ [HL(CrO_4_)]^−^	3.55 (5)
H_2_L^2+^ + CrO_4_^2−^ ⇆ [H_2_L(CrO_4_)]	3.75 (5)
H_2_L^2+^ + HCrO_4_^−^ ⇆ [H_2_L(HCrO_4_)]^+^	3.81 (3)
Hg^2+^ + L ⇆ [HgL]^2+^	16.47 (3)
Hg^2+^ + HL^+^ ⇆ [HgHL]^3+^	13.06 (5)
[HgL]^2+^ + OH^−^ ⇆ [HgL(OH)]^+^	3.87 (4)
[HgL(OH)]^+^ + OH^−^ ⇆ [HgL(OH)_2_]	3.24 (7)
H_2_L^2+^ + HgCl_4_^2−^ ⇆ [H_2_L(HgCl_4_)]	8.41 (6)
H_3_L^3+^ + HgCl_4_^2−^ ⇆ [H_3_L(HgCl_4_)]^+^	8.75 (2)

* Values in parentheses are standard deviations in the last significant figure.

**Table 2:** Stability constants of FN-HBPEI/anion complexes in solution (0.1 M KCl, 298.0 K).

Equilibrium	Log *K*
HL_2_^+^ + AsO_4_^3−^ ⇆ [HL_2_(AsO_4_)]^2−^	6.84 (3)
HL^+^ + HAsO_4_^2−^ ⇆ [HL(HAsO_4_^2−^)]^−^	2.13 (6)
H_2_L^2+^ + HAsO_4_^2−^ ⇆ [H_2_L(HAsO_4_)]	3.99 (4)
HL_2_^+^ + PO_4_^3−^ ⇆ [HL_2_(PO_4_)]^2−^	7.15 (4)
HL^+^ + HPO_4_^2−^ ⇆ [HL(HPO_4_^2−^)]^−^	2.92 (7)
H_2_L^2+^ + HPO_4_^2−^ ⇆ [H_2_L(HPO_4_)]	3.58 (5)
HL_2_^+^ + CrO_4_^2−^ ⇆ [HL_2_(CrO_4_)]^−^	4.18 (9)
HL^+^ + CrO_4_^2−^ ⇆ [HL(CrO_4_)]^−^	3.62 (6)
H_2_L^2+^ + CrO_4_^2−^ ⇆ [H_2_L(CrO_4_)]	3.88 (5)
H_2_L^2+^ + HCrO_4_^−^ ⇆ [H_2_L(HCrO_4_)]^+^	4.25 (2)
Hg^2+^ + L ⇆ [HgL]^2+^	17.17 (5)
Hg^2+^ + HL^+^ ⇆ [HgHL]^3+^	14.23 (6)
[HgL]^2+^ + OH^−^ ⇆ [HgL(OH)]^+^	3.57 (2)
[HgL(OH)]^+^ + OH^−^ ⇆ [HgL(OH)_2_]	2.01 (8)
H_2_L^2+^ + HgCl_4_^2−^ ⇆ [H_2_L(HgCl_4_)]	---
H_3_L^3+^ + HgCl_4_^2−^ ⇆ [H_3_L(HgCl_4_)]^+^	---

**Table 3:** Textural parameters.

Sample	S (BET) (m^2^g^−1^)	V_0_ (N_2_) (cm^3^g^−1^)	L_0_ (N_2_) (nm)	Sext (m^2^g^−1^)	V_0_ (CO_2_) (cm^3^g^−1^)	L_0_ (CO_2_) (nm)
FN-HBPEI	77	0.028	1.6	6.8	0.065	0.5
F	1426	0.561	1.3	39.2	0.315	0.8

S(BET), specific surface (BET equation): Calculated from the N_2_ adsorption isotherms at 77 K. V_0_(N_2_), pore volume (Dubinin–Radushkevich equation): Calculated from the N_2_ adsorption isotherms at 77 K. L_0_(N_2_), mean pore width (Dubinin–Radushkevich equation): Calculated from the N_2_ adsorption isotherms at 77 K. S_ext_, specific external surface (alpha method): Calculated from the N_2_ adsorption isotherms at 77 K. V_0_(CO_2_), pore volume (Dubinin–Radushkevich equation): Calculated from the CO_2_ adsorption isotherms at 273 K. L_0_(CO_2_), mean pore width (Dubinin–Radushkevich equation): Calculated from the CO_2_ adsorption isotherms at 273 K.

**Table 4:** Structural parameters of HBPEI derivatives.

Proton Species of HBPEI	Dimensional Sizes (nm)			Min–Max Area Interval (nm^2^)	Molecular Volume (nm^3^)	Molecular Deviation (%)
	X	Y	Z			
L	2.26	1.00	2.32	2.26–5.23	5.25	100
HL^+^	2.16	0.86	2.48	1.86–5.36	4.62	88
HL^2+^	2.05	0.87	2.22	1.78–4.55	3.97	76
HL^3+^	1.90	1.38	3.09	2.63–5.88	8.14	155

## Data Availability

The original contributions presented in this study are included in the article; further inquiries can be directed to the corresponding author.

## Supplementary Materials

The following supporting information can be downloaded at https://www.mdpi.com/article/10.3390/polym17060786/s1. Figure S1. TGA analysis of HBPEI and FN-HBPEI; Figure S2. F, FN, FN-OMe and FN-HBPEI NMR spectra; Figure S3. N(1s) XPS spectra of (a) HBPEI and (b) FN-HBPEI; Figure S4. H_3_O^+^ adsorption isotherms, measured at 298 K and ionic strength 0.1 M KCl, of F, FN, FN-OMe and FN-HBPEI; Figure S5. UV spectra of an L/HgCl_4_^2−^ mixture in aqueous solution ([L]/[HgCl_4_^2−^] = 1/1, [L] = 5 × 10^−5^ M) in the 1–13 pH range; Figure S6. Species distribution of HBPEI/anion systems; Figure S7. Species distribution of FN-HBPEI/anion systems; Table S1. Textural data; Table S2. Langmuir parameters.

## Abbreviations

The following abbreviations are used in this manuscript:

HBPEI	Hyperbranched polyethyleneimine
AC	Activated carbon
F	Commercial activated carbon (Filtracarb)
FN	Activated carbon (F) oxidized with HNO_3_
FN-OME	Activated carbon (FN) esterified
FN-HBPEI	Hybrid material based on HBPEI molecules bonded to FN-OMe
L	Complexing HBPEI unit based on triethylenetriamine fragments (L= –NH(CH_2_)_2_–N[–(CH_2_)_2_NH_2_]–(CH_2_)_2_–)
HL	Protonated complexing HBPEI unit
