# Supplementary material for: Carbon-Supported Hyperbranched Polyethyleneimines: Exploring into Polyamine/Anion Interactions to Design Efficient Polymer-Based Energy and Scavenger Materials
Source: Polymers (Basel). 2025 Mar 15;17(6):786. doi: 10.3390/polym17060786 (PMC11944631; doi:10.3390/polym17060786)
Supplement: Supplementary file 1 [file polymers-17-00786-s001.zip › polymers-3518759-supplementary.pdf]

Supporting information

Carbon-supported hyperbranched polyethyleneimines: insights into polyamine / anion interactions to design efficient polymer-based energy and scavenger materials

Antonio Peñas-Sanjuán[1*], Celeste García-Gallarín[1], María L. Godino-Salido[1], Rafael López-Garzón[1], Michele Melchionna[2], and Manuel Melguizo[1]

1. Textural data for carbon based material (F, FN, FN-OMe and FN-HBPEI).
2. TGA analysis of HBPEI and FN-HBPEI
3. Solid state ^{13}C-NMR DP-MAS spectra of the carbon based materials (F, FN, FN-OMe and FN-HBPEI).
4. XPS characterization
5. H_3O^+ adsorption isotherms obtained by titration
6. Langmuir parameters
7. UV spectra of L / $HgCl_4^{2-}$ mixture in aqueous solution at different pH values
8. Species distribution plots of HBPEI / anion and FN-HBPEI / anion systems.

1) Textural data

Table S 1. Textural data

Sample	S (BET) [m^2g^{-1}]	V_0 (N_2) [$cm^3 \cdot g^{-1}$]	$L_0(N_2)$ [nm]	S_{ext} [m^2 g^{-1}]	V_0 (CO_2) [$cm^3 \cdot g^{-1}$]	$L_0(CO_2)$ [nm]
**F**	1426	0.561	1.3	39.2	0.315	0.8
**FN**	691	0.279	1.3	7.8	0.157	0.6
**FN-OMe**	661	0.234	1.2	9.7	0.154	0.6
**FN-HBPEI**	77	0.028	1.6	6.8	0.065	0.5

S(BET). Specific surface (BET equation). Calculated from the N_2 adsorption isotherms at 77 K
$V_0(N_2)$. Pore volume (Dubinin-Radushkevich equation). Calculated from the N_2 adsorption isotherms at 77K
$L_0(N_2)$. Mean pore width (Dubinin-Radushkevich equation). Calculated from the N_2 adsorption isotherms at 77 K
$S_{ext.}$ Specific external surface (alpha method). Calculated from the N_2 adsorption isotherms at 77 K
V_0 (CO_2). Pore volume (Dubinin-Radushkevich equation). Calculated from the CO_2 adsorption isotherms at 273 K.
L_0 (CO_2). Mean pore width (Dubinin-Radushkevich equation). Calculated from the CO_2 adsorption isotherms at 273 K.

2) TGA analysis of HBPEI and FN-HBPEI

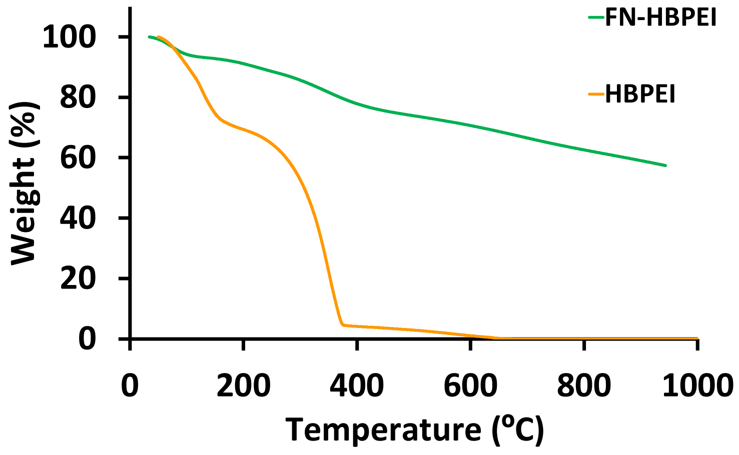

Figure S 1. TGA analysis of HBPEI and FN-HBPEI

3) Solid state ^{13}C-NMR DP-MAS spectra.

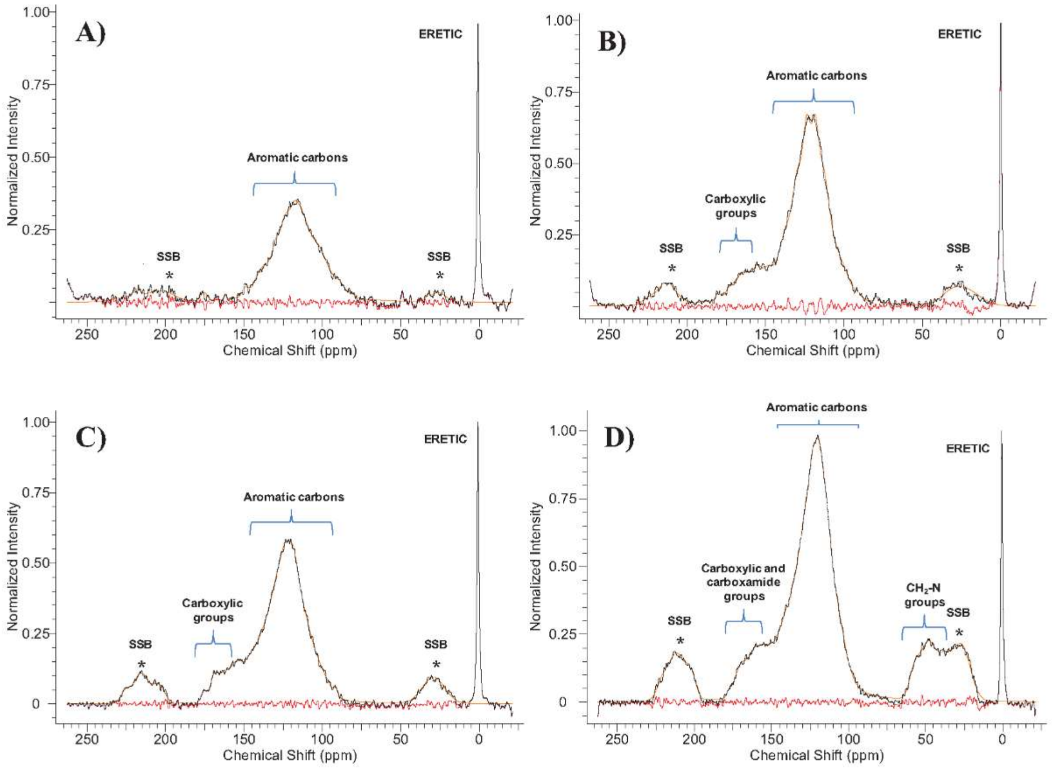

Figure S 2. A) F, B) FN, C) FN-OMe, D) FN-HBPEI NMR spectra. Electronic reference (ERETIC, 0 ppm) and spinning sidebands of the central signal (SSB, 205.86 ppm and 28.16 ppm) included in the spectrum.

4) XPS characterization

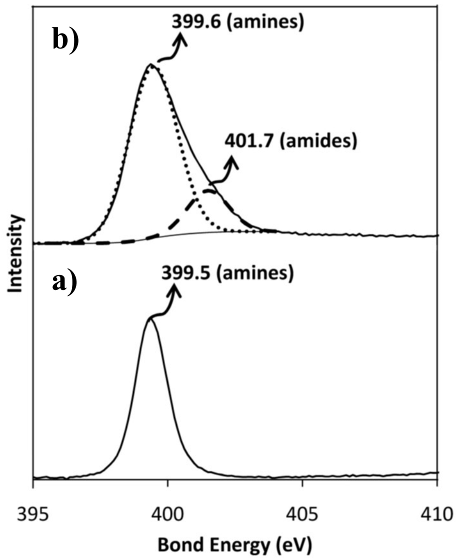

Figure S 3. N(1s) XPS spectra of: a) HBPEI and b) FN-HBPEI, where deconvolution of the amine and amide components yields an amine/amide ratio of 83:17.

5) H_3O^+ adsorption isotherms obtained by titration

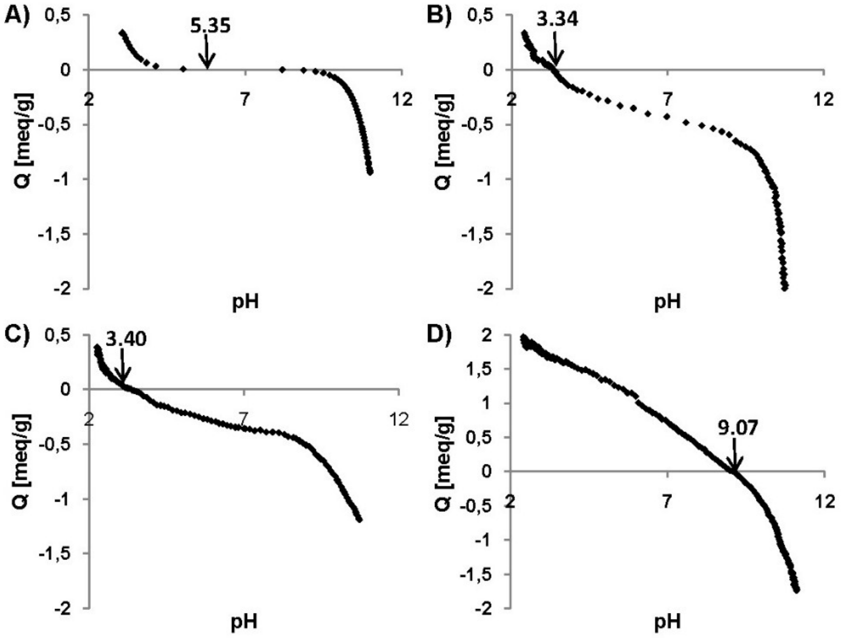

Figure S 4. H_3O^+ adsorption isotherms, measured at 298K and ionic strength 0.1M KCl, of A) F, B) FN, C) FN-OMe, D) FN-HBPEI.

6) Langmuir parameters

Table S 2. Langmuir parameters

Metal	pH of adsorption	X_m (mmol / g)	R^2
CrO_4^{2-}	7.5	0.15	0.97
PO_4^{3-}	6.7	0.13	0.98
AsO_4^{3-}	6.7	0.16	0.98
$HgCl_4^{2-}$	3.0	0.16	0.95

7) UV spectra of L / $HgCl_4^{2-}$ mixture in aqueous solution at different pH values

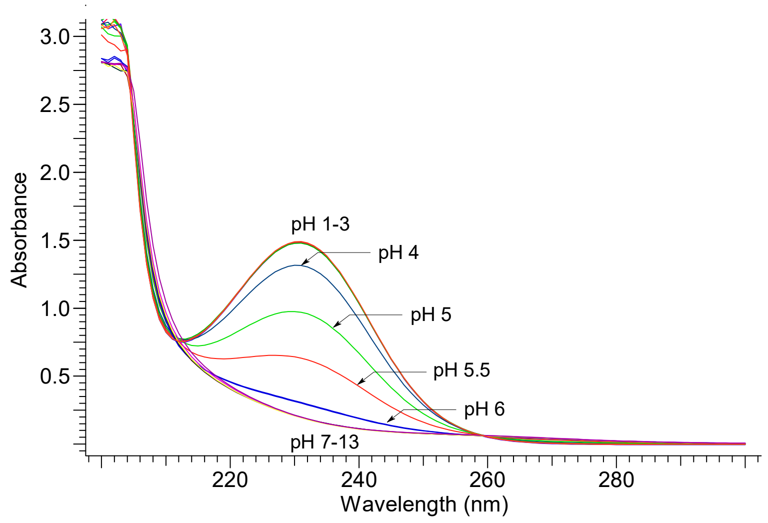

Figure S 5. UV spectra of a L / $HgCl_4^{2-}$ mixture in aqueous solution ([L] / [$HgCl_4^{2-}$] = 1/1, [L] = 5 x 10^{-5} M) in the 1-13 pH range

8) Species distribution plots of HBPEI / anion and FN-HBPEI / anion systems.

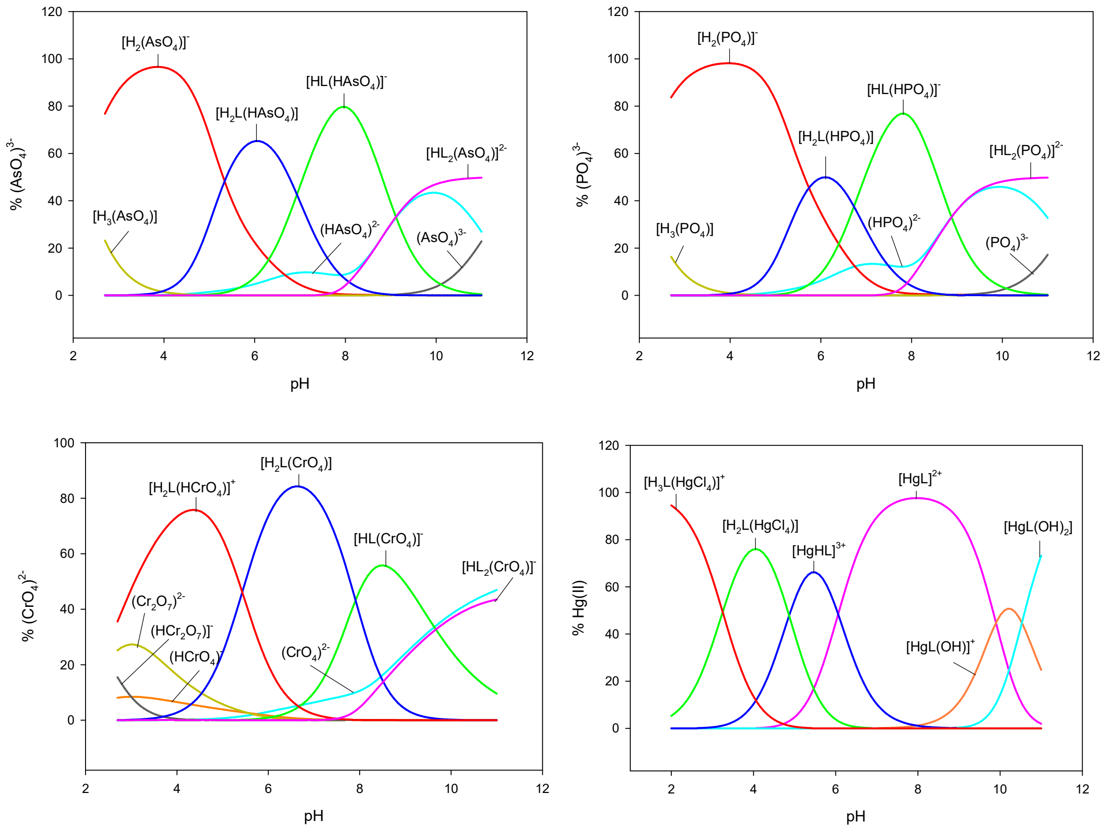

Figure S 6. Species distribution of HBPEI / anion systems

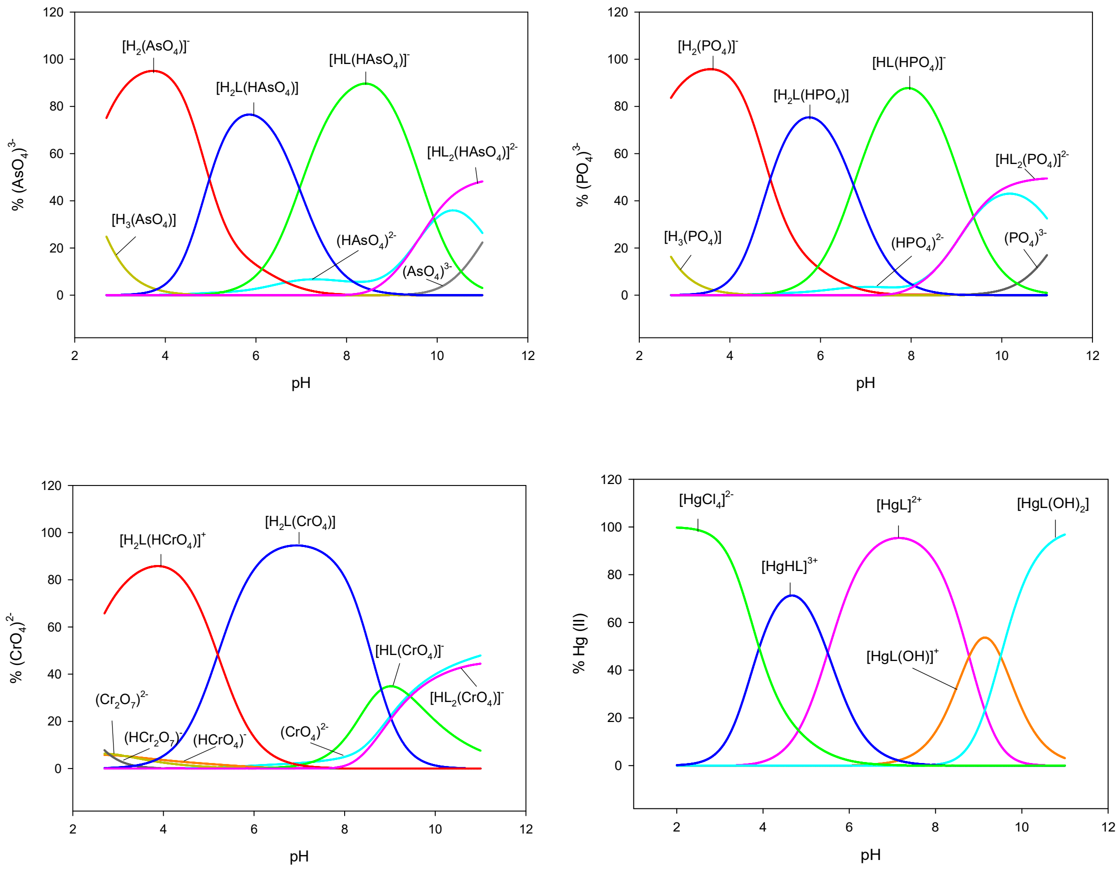

Figure S 7. Species distribution of FN-HBPEI / anion systems
